# Theoretical and Experimental Studies on the Evidence of 1,3-β-Glucan in Marennine of *Haslea ostrearia*

**DOI:** 10.3390/molecules28155625

**Published:** 2023-07-25

**Authors:** Muhammad Yusuf, Umi Baroroh, Rina Fajri Nuwarda, Fiddy Semba Prasetiya, Safri Ishmayana, Mia Tria Novianti, Taufik Ramdani Tohari, Ari Hardianto, Toto Subroto, Jean-Luc Mouget, Pamela Pasetto

**Affiliations:** 1 Department of Chemistry, Faculty of Mathematics and Natural Sciences, Universitas Padjadjaran, Sumedang 45363, Indonesia; m.yusuf@unpad.ac.id (M.Y.); ishmayana@unpad.ac.id (S.I.); a.hardianto@unpad.ac.id (A.H.); t.subroto@unpad.ac.id (T.S.); 2 Research Center for Molecular Biotechnology and Bioinformatics, Universitas Padjadjaran, Bandung 40133, Indonesia; umibaroroh@stfi.ac.id (U.B.); miatria17@gmail.com (M.T.N.); taufikramdanitohari1@gmail.com (T.R.T.); 3 Department of Biotechnology Pharmacy, Indonesian School of Pharmacy, Bandung 40266, Indonesia; 4 Department of Pharmaceutical Analysis and Medicinal Chemistry, Faculty of Pharmacy, Universitas Padjadjaran, Sumedang 45363, Indonesia; rina.nuwarda@unpad.ac.id; 5 Research Center for Biosystematics and Evolution, Research Organization for Life Sciences and Environment, National Research and Innovation Agency (BRIN), Cibinong 16911, Indonesia; fidd001@brin.go.id; 6 Laboratoire Biologie des Organismes, Stress, Santé, Environnement (BiOSSE), Le Mans Université, Avenue Olivier Messiaen, 72085 Le Mans, France; jean-luc.mouget@univ-lemans.fr; 7 Institut des Molécules et Matériaux du Mans (IMMM), UMR CNRS 6283, Le Mans Université, Avenue Olivier Messiaen, 72085 Le Mans, France

**Keywords:** marennine, *Haslea ostrearia*, endo-1,3-β-glucanase, β-glucan, hydrolysis, computational study

## Abstract

Marennine, a blue pigment produced by the blue diatom *Haslea ostrearia*, is known to have some biological activities. This pigment is responsible for the greening of oysters on the West Coast of France. Other new species of blue diatom, *H. karadagensis*, *H. silbo* sp. inedit., *H. provincialis* sp. inedit, and *H. nusantara*, also produce marennine-like pigments with similar biological activities. Aside from being a potential source of natural blue pigments, *H. ostrearia*-like diatoms present a commercial potential for the aquaculture, food, cosmetics, and health industries. Unfortunately, for a hundred years, the exact molecular structure of this bioactive compound has remained a mystery. A lot of hypotheses regarding the chemical structure of marennine have been proposed. The recent discovery of this structure revealed that it is a macromolecule, mainly carbohydrates, with a complex composition. In this study, some glycoside hydrolases were used to digest marennine, and the products were further analyzed using nuclear magnetic resonance (NMR) and mass spectroscopy (MS). The reducing sugar assay showed that marennine was hydrolyzed only by endo-1,3-β-glucanase. Further insight into the structure of marennine was provided by the spectrum of ^1^H NMR, MS, a colorimetric assay, and a computational study, which suggest that the chemical structure of marennine contains 1,3-β-glucan.

## 1. Introduction

Several marine organisms are beautifully colored due to the variety of pigments contained in their cells [1,2]. These pigments show diversity in color and may display a great variety imitating certain organisms. In microalgae, for instance, chlorophyll is identical to the green color that is extremely abundant in green algae, such as *Chlorella vulgaris*, *Spirulina subsalsa*, *Nannochloropsis oculata*, etc. [3,4]. However, the blue color is so scarcely distributed that it can be observed only in a few organisms, for instance, in the terrestrial bacteria *Pseudomonas aeruginosa* that produces pyocyanin [5,6], a blue pigment that possesses antibiotic activities [7], and *Pantoea agglomerans* has recently been shown to produce a novel “deep blue” pigment [8].

The blue pigments are scarcely found in marine bacteria. Only a few marine bacteria are able to synthesize blue pigments [9], such as several species from the genus *Rheinheimera* that produces glaukothalin [10,11] and the genus *Phaeobacter* that produces the pigment indigoidine [8]. On the other hand, blue pigments can be observed in the photosynthetic organisms, for instance, in the photosynthetic eukaryotes, such as *Aurearena cruciata* in its senescent stage [12] and in the pennate diatom *Haslea ostrearia* (Bacillariophyceae) during its exponential phase of growth and aging [13].

The present study focused on the pennate diatom *H. ostrearia* and its blue pigment marennine that has long been known as the cause of the oyster greening phenomenon in the Atlantic Coast of France [14,15]. Recent studies revealed that other *Haslea* species either from the Northern or Southern Hemisphere have a similar peculiarity in producing a blue marennine-like pigment, such as *H. karadagensis*, *H. provincialis*, *H. silbo*, and *H. nusantara* [14,16,17]. Several of these species display similar biological activity compared to that of *H. ostrearia*, such as antibacterial, antiviral, antiproliferative, antioxidant, and antifungal activities [18,19,20].

Despite its many biological activities, the molecular structure of marennine is still unknown. A successful elucidation of the structure would support the development of this pigment in many fields. The earlier hypothesis that marennine is a type of metallic salt was instantly rejected [21,22,23,24]. Moreover, the hypotheses that suggest that marennine is closely related to carotenoids [25] or chlorophylls [26,27,28,29,30,31] are also still unproven. Additionally, other authors tried to associate marennine with cyanobacterial pigments, hence suggesting a protein nature for this hydro-soluble molecule [32,33,34]. Some others proposed that marennine could be an anthocyanin with respect to stress and pigment accumulation in cells [35]. Other studies related marennine to a mixture of different types of macromolecules [36] or a polyphenolic nature [37]. The very recent study in 2023 conducted by Zebiri et al. using NMR spectroscopy found that marennine’s spectra mainly consist of carbohydrate complexes. Galactose, xylose, rhamnose, and fucose were the most abundant monosaccharides built after hydrolysis [38]. Interestingly, glucose, which predominates the polysaccharides in other diatoms [39], was not found in marennine. Considering the previous study was conducted using chemical hydrolysis, we were intrigued to explore the possibility of glucose existence in marennine using different approaches, e.g., enzymatic hydrolysis and a colorimetric assay. It has been shown that marennine exists in two slightly different forms, intracellular and extracellular (IMn and EMn, respectively) [40], and that for both forms, the color changes with pH from blue (acidic pH) to green (basic pH) [37]. It was suggested that marennine could be a complex macromolecule composed of a polyglycosidic backbone and a small organic molecule associated as a chromophore [14,41]. To model the possibility of a glycan component in marennine, we choose laminarin, a naturally occurring polysaccharide that can be detected using aniline blue dye. Laminarin is a linear β-1,3-glucan with some β-1,6 inner-strand linkage and branch points. They can form a single- or triple-helix structure that increases its thermal stability [42]. It is noted that marennine has a good thermal stability [14]. Another dye that can be used to detect β-1,3-glucan is congo red. The interaction between congo red dye and β-1,3-D-glucan has been studied and shows a bathochromic shift from 488 to 516 nm in the visible adsorption maximum of congo red [43].

In this study, aniline blue and congo red were used as pigments to study the molecular interaction in β-1,3-glucan using computational methods and a colorimetric assay, respectively. The model of laminarin and aniline blue was simulated by molecular dynamics simulation to predict the possible mode of binding and a model hypothesis of marennine components, while congo red was used to detect the bathochromic shift in the spectrophotometer using a colorimetric assay. Furthermore, since an enzymatic reaction is able to catalyze reactions with a very high degree of specificity, we used carbohydrate hydrolysis enzymes to cut selectively targeted glycosidic bonds to help in the characterization of the entire molecular structure, starting from the smaller fragments. Pouvreau et al. [37] tested four enzymes able to selectively cut α- or β-glycosidic linkages using α-d-glucosidase, α-d-galactosidase, α-d-mannosidase, and β-d-glucosidase, but they did not catalyze any reaction. Gastineau et al. [41] tried to degrade marennine using a cellulase cocktail produced by *Aspergillus niger*, and interestingly, it showed a positive reducing sugar result. The ^1^H-NMR spectrum of the crude reaction supernatant after incubation presented some peaks characteristic of glucose. This finding led us to investigate more using various glycoside hydrolases. Therefore, this study aimed at investigating the chemical structure of marennine by using enzymatic assays, nuclear magnetic resonance, a colorimetric assay, and mass spectrometry (MS). The molecular interaction between laminarin and anilin blue was also provided as a model molecule to study marennine’s structure as well as congo red with laminarin. The hypotheses regarding the possible glycan existence of this pigment will be discussed in this article.

## 2. Results

### 2.1. Computational Study

Laminarin was chosen as the model of part of marennine because, in this work, the sample was cleaved by 1,3-β-glucanase, which specifically recognized the β-Glc-(1,3)-Glc bond. One of the known 1,3-β-glucans is laminarin. This modeling effort was performed because, previously, acidic hydrolysis of marennine resulted in monomers, e.g., β-galactose, β-fucose, and β-xylose [38]. Therefore, it is difficult to predict the polysaccharide model from the monomers’ information.

**Figure 1:**
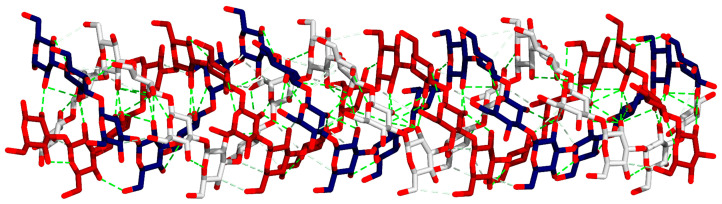
Triple-helix structure of laminarin; each chain is colored differently in the red, white, and blue backbone stick model. Hydrogen bonds are depicted with green dashed lines. Numerous hydrogen bonds support the stability of the triple-helix structure of laminarin.

**Figure 2:**
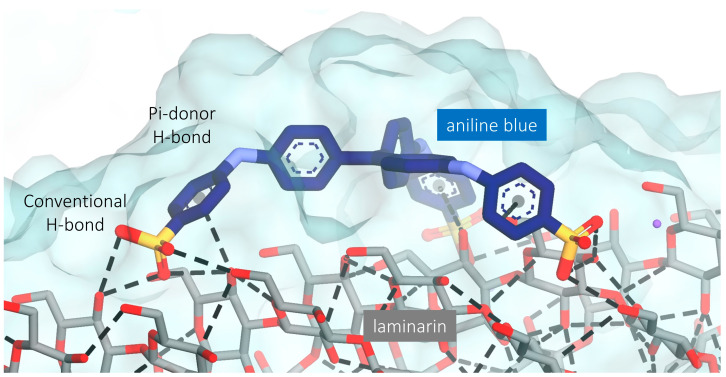
Molecular interaction of laminarin and aniline blue after the docking study. Hydrogen bonds are depicted with a dashed line between the H-bond donor and acceptor. The dashed line between the center of the ring in aniline blue and the hydroxyl group of laminarin represents the Pi-donor hydrogen bond.

The model structure of laminarin showed a lot of intermolecular and intramolecular hydrogen bonds, which suggested a compact structure (Figure 1). This study was conducted to predict the possible mode of binding between laminarin and aniline blue. From molecular docking, aniline blue non-covalently bound to the wide surface of laminarin. The interactions between laminarin and aniline blue are Pi-donor H-bond, electrostatic interaction, and conventional H-bond. It appears that aniline blue needs the triple-helical form of laminarin to bind (Figure 2). The calculated free energy of binding using MM/GBSA was −9 kcal/mol; this value was comparable to the nanomolar strength of non-covalent binding.

### 2.2. Enzymatic Reactions

**Figure 3:**
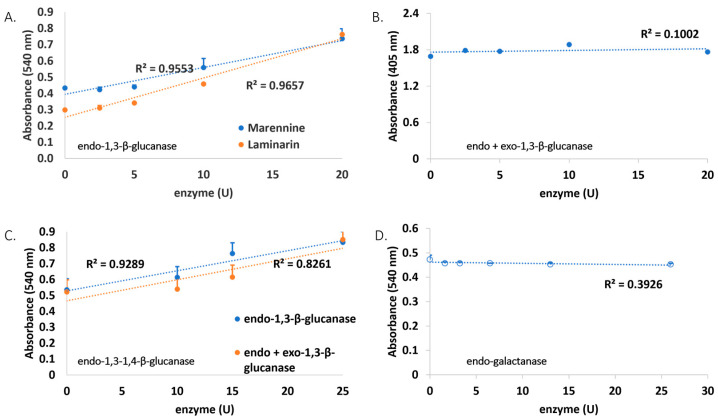
The absorbance values at 540 nm of (**A**) laminarin and marennine solutions after hydrolysis with endo-1,3-b-glucanase, (**B**) marennine after hydrolysis with endo-1,3-b-glucanase followed by exo-1,3-b-glucanase, (**C**) marennine with endo-1,3-1,4-b-glucanase, and (**D**) marennine with endo-galactanase.

All marennine enzymatic digestion products were assayed for their reducing sugar content using the DNS (3,5-Dinitrosalicylic acid) method. Laminarin, a storage glucan found in brown algae, was used as a positive control, being a substrate of endo-1,3-β-glucanase. Both marennine and laminarin showed positive results with the enzyme. Hence, it is indicated that marennine has a β-glucan component similar to laminarin. The Wilcoxon signed-rank test was used to test whether both data were different, and the results showed that they were not significantly different (*p* > 0.05). Exo-1,3-β-glucanase was also tested after hydrolysis with endo-1,3-β-glucanase to investigate whether it contains β-glucan at the terminal end. The negative result suggests that marennine does not have an accessible β-glucan at the terminal end. It was also supported by the *p* > 0.05, which showed that both treatments were not significantly different. Lichenase, an endo-1,3-1,4-β-glucanase with different bond specificity, was also tested to probe another linkage type. As a result, the absorbance change was not detected, which suggests that the linkage of endo-1,3-1,4-β-glucan is absent in marennine. All the previous enzyme assays were performed to check the presence of glucan. Furthermore, endo-galactanase was used to check for the existence of galactan in marennine. This test gave a negative result, indicating that marennine is not composed of galactan (Figure 3). However, this suggestion is not denying the possible presence of galactose as part of the carbohydrate chain.

**Figure 4:**
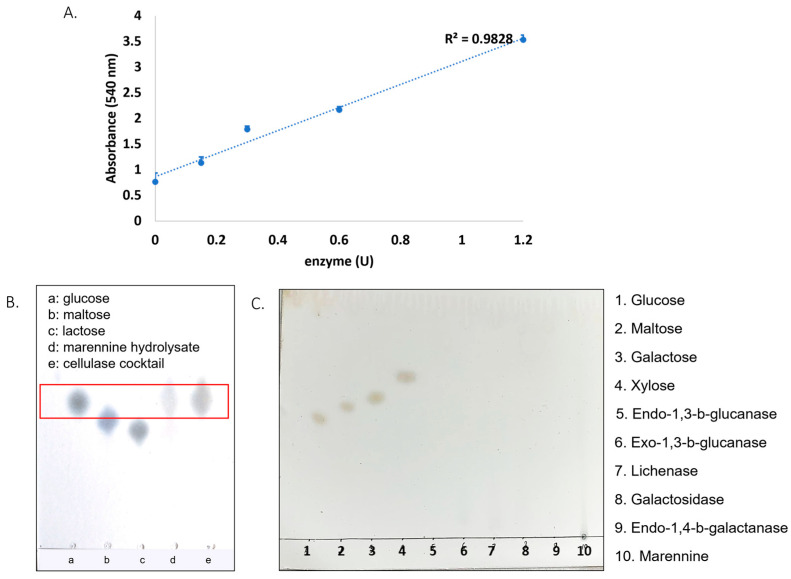
(**A**) Marennine absorbance after the reaction with the cocktail enzyme. (**B**) TLC analysis of cellulase cocktail enzyme and references, and (**C**) TLC analysis of the rest of the enzymes (lanes 5–9).

From these results, it can be hypothesized that marennine contains 1,3-β-glucan as part of its complex structure. A cellulase cocktail enzyme was also tested on marennine using the DNS assay. The absorbance showed a linear line, indicating that the hydrolysis reaction occurred (Figure 4A). For further analysis, thin-layer chromatography (TLC) was performed using glucose, maltose, and lactose as controls. However, the marennine hydrolysate had the same R_f_ as glucose and the enzyme cocktail (Figure 4B). This result alerted us about the existence of glucose in the enzyme preparations. For this reason, the presence of free glucose in the other enzymes was tested using TLC. As a result, free glucose was not observed in the rest of the enzymes (Figure 4C, lanes 5–9, using lanes 1–4 as controls).

### 2.3. Spectroscopic Analysis

**Figure 5:**
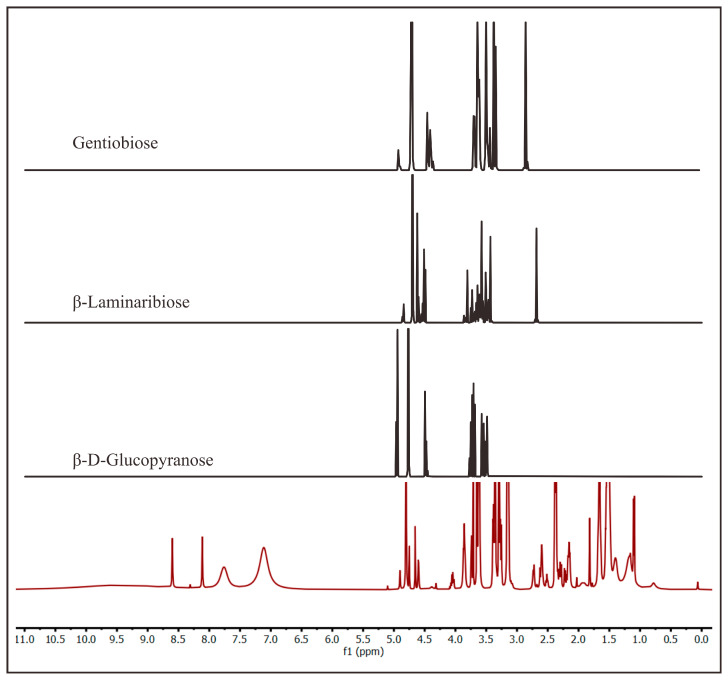
Comparison of the ^1^H-NMR spectra of the dialysate of marennine after hydrolysis (red line) with endo-1,3-β-glucanase (100–500 Da cutoff) and other references of β-glucan obtained in D_2_O [44].

Since the marennine showed positive results with endo-1,3-β-glucanase, ^1^H NMR and MS were used to probe for the presence of 1,3-β-glucan in marennine. Marennine was hydrolyzed with endo-1,3-β-glucanase overnight; then, it was dialyzed with a 100–500 Da cutoff membrane. The permeate was evaporated to obtain a concentrated sample. Due to the size of the membrane pores, the expected compounds from this sample were monosaccharides and disaccharides. The ^1^H-NMR spectrum showed peaks at 3.5, 4.7, and 8.02 ppm, corresponding to glycerol, residual H_2_O in D_2_O, and formic acid, respectively. Glycerol was present in the enzyme stock. A purification with dialysis to remove the glycerol was attempted, but it only partially eliminated it. Finally, the target sample concentration was considerably low. Therefore, the MestReNova program was used to process the ^1^H-NMR spectra of this sample. The program removed the peak of glycerol, H_2_O, and formic acid, and the intensity threshold was decreased to under 300. Many peaks from 1 to 5 ppm and 7 to 9 ppm appeared, and those from 3 to 5 ppm are typical for sugars. These synthetic peaks were compared with known peaks for the other β-glucans, i.e., β-d-glucopyranose, β-laminaribiose, and gentiobiose [44], The comparison showed similar characteristics (Figure 5) indicated by the appearance of the signal of β–anomeric protons at 4.5 ppm. The spectrum of β-laminaribiose is similar to that of the hydrolysate of marennine, although further confirmation on the structure is required. Whereas for ^13^C NMR, the spectrum was dominated by glycerol and could not be further processed with the same method as ^1^H NMR. Moreover, the 2D NOESY NMR of this sample showed only glycerol due to the low concentration of marennine even after evaporation. The original ^1^H-NMR and ^13^C-NMR spectra are provided in the Supplementary Materials.

**Figure 6:**
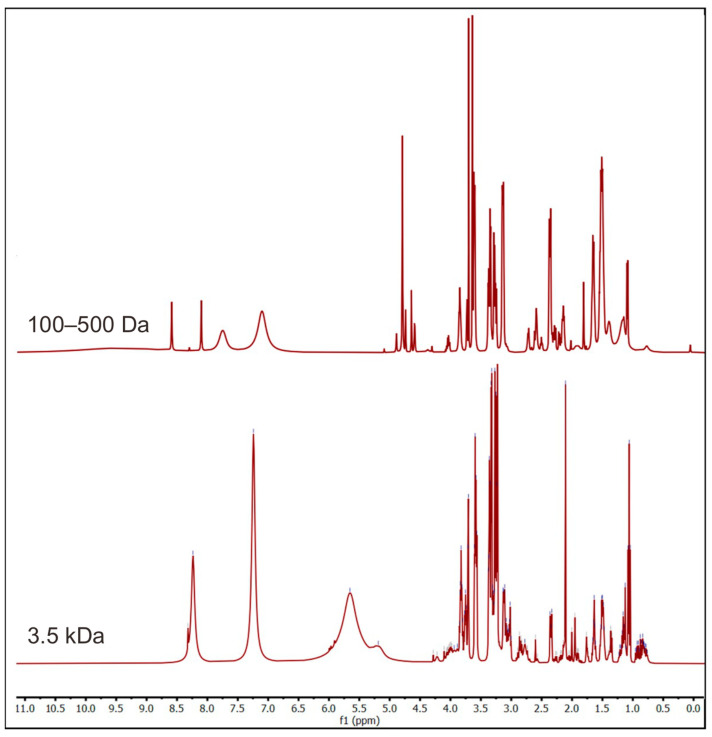
Comparison of ^1^H-NMR spectra of marennine dialysate with 100–500 Da and 3.5 kDa membrane cutoffs.

**Figure 7:**
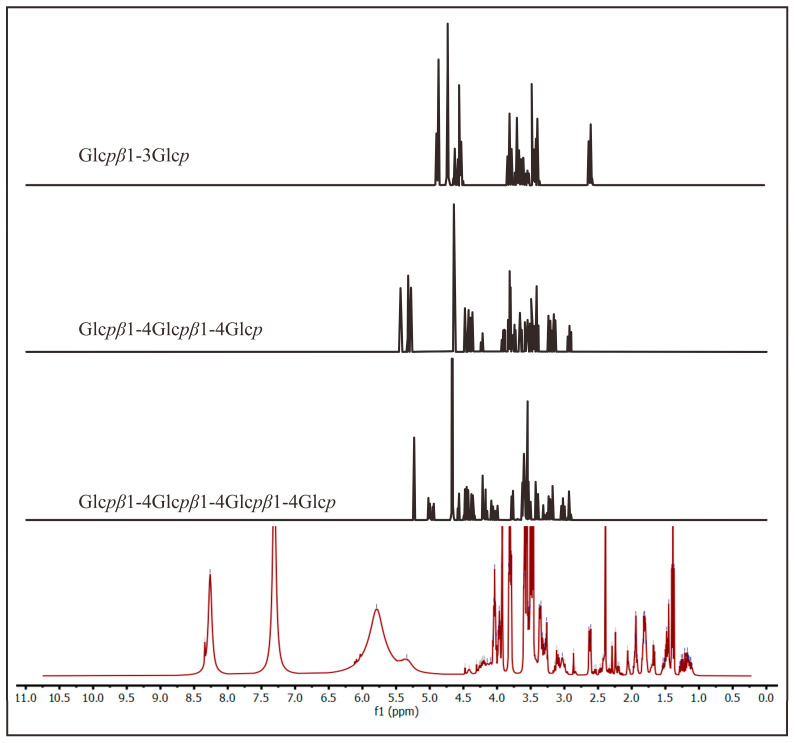
Comparison of ^1^H-NMR spectra of the dialysate of marennine with the 3.5 kDa cutoff (red line) with other references of β-glucan [44].

Moreover, another dialysis membrane with a 3.5 kDa cutoff was used to obtain bigger fragments. Similar processing of the ^1^H-NMR spectra was performed on the sample, and it is shown in Figure 6. The new spectrum showed peaks at 3–5 ppm like the previous one. The difference between the two spectra was at 4.5–9.0 ppm. The second spectrum was also compared with the other disaccharide, trisaccharides, and tetrasaccharides (Figure 7). It is noted that the peak at 5.5 ppm in the new spectrum was also found in trisaccharides, indicating the presence of oligosaccharides in the permeate within the 3.5 kDa cutoff. In addition, the peak at 7–8.5 ppm was suggested to be the aromatic form of the chromophore, the blue part of marennine.

**Figure 8:**
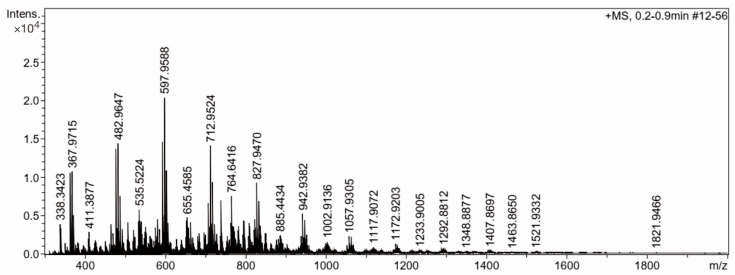
Mass spectroscopy spectrum of marennine after hydrolysis with the mixed enzyme.

Lastly, marennine was incubated with a mixed enzyme composed of endo-1,3-β-glucanase, exo-1,3-β-glucanase, lichenase, galactosidase, and endo-1,4-β-galactanase. The sample was dialyzed with a 3.5 kDa membrane cutoff, and the resulting permeate was evaporated. Furthermore, the sample was analyzed using MS spectroscopy. Interestingly, a regular pattern of the MS spectrum with *m*/*z* 115 differences is shown in Figure 8. The mass of *m*/*z* 115 is known to be the glucose fragment from the 1,3-type of β-glucan [45]. According to Berman and colleagues, this molecule was the fragmentation of glucose by the loss of the small neutral molecule formaldehyde [45]. The list of *m*/*z* values of the mass spectroscopy spectrum is presented in Table 1.

### 2.4. Colorimetric Assay

**Figure 9:**
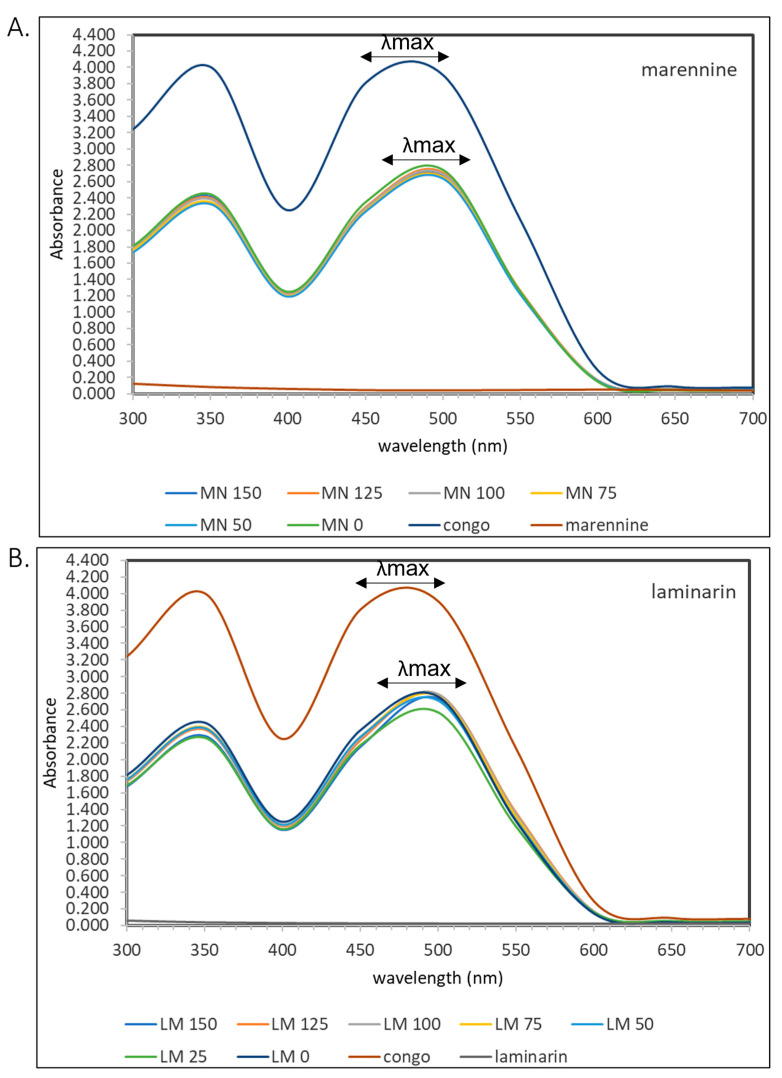
UV–Vis spectra in the range of 300–700 nm in (**A**) congo red alone and with marennine in a range of 50–150 μg/mL and (**B**) congo red alone and with laminarin in a range of 25–150 μg/mL.

Congo red is a molecule with a formula of C_32_H_22_N_6_O_6_S_2_Na_2_ and consists of a linear molecule. Semedo et al. studied the effect of congo red in several mushrooms that contained β-d-glucans. It showed that a bathochromic shift >20 nm was detected in the UV-Vis spectrophotometer [43]. The same method was applied to marennine and laminarin and showed a similar shift (Figure 9). A mixture of congo red with marennine and laminarin in a range of 25–150 μg/mL was quantified at 300–700 nm in a UV–Vis spectrophotometer. It showed a bathochromic shift of approximately 15 nm for both, strengthening the presence of 1,3-β-glucan in marennine.

## 3. Discussion

The definition of the chemical structure of marennine is quite challenging due to its high molecular weight of 9.8 kDa determined once with mass spectrometry [37] and confirmed with two-dimensional NMR [14]. Previously, marennine was hypothesized to be a carotenoid [25], a chlorophyll degradation product [26,27,28,29,30,31], an anthocyanin [35], and a polyphenolic compound [37]. An NMR study by Gastineau et al. in 2018 suggested a new hypothesis for the chemical structure [41]. The presence of protons and carbon in the chemical shift range typical of a glycosidic ring indicated that part of the structure is glycosidic. A very recent study conducted by Zebiri et al. found from NMR spectra after chemical hydrolysis that marennine consists of galactose, xylose, mannose, rhamnose, and fructose without glucose [38]. Our study examined these findings by using a different approach for marennine hydrolysis. Some carbohydrate enzymes were used followed by computational analysis and a calorimetric assay to support our finding that part of marennine also consists of 1,3-β-glucan.

1,3-β-glucans are natural polysaccharides with unique pharmacological properties and the ability to form single- and triple-helical structures [46]. The triple-helical β-1,3-glucan structure can affect living organisms’ immune and physiological functions [47,48]. Another pharmacological effect of triple-helical β-1,3-glucan is that it can enhance the immune system, thus leading to several effects, such as antitumor, antibacterial, and wound-healing activities [42]. This corresponds to the biological activities of marennine, such as antibacterial, antiviral, antifungal, antiproliferative, and antioxidant properties [14,18,49]. For this reason, the β-glucan part of marennine is suggested to form a triple-helix structure. Marennine can easily be precipitated by the addition of NaOH. This phenomenon also occurred when triple-helical glucan was treated with NaOH. Its conformer was partially opened due to the deprotonation, decreasing its solubility [42]. After neutralizing with acid, the partially opened conformer gradually reverts to the triple-helical structure. The precipitated marennine by NaOH was also easily resolubilized by adding formic acid. In addition, the endo-1,3-β-glucanase used in this experiment naturally cleaved the triple-helical glucan. The enzyme has a specific cavity that is large enough to hold the bulky structure of the substrate [48]. Therefore, the negative result for the other enzymes might be due to the inability of the active site to accept the triple-helix conformer, although the type of linkage might be present. The preliminary circular dichroism experiment was performed on a marennine solution in water (Jasco Spectropolarimeter J-810) between 195 and 260 nm. However, no significant signal was detected—further study to modify the polysaccharide by adding -COOH groups to monitor a CD signal is required.

It is worth noting that the enzyme has high activity when its substrate only contains β-1,3 glucan, not the other linkages [48]. Laminarin, a linear β-1,3-glucan with some β-1,6 inner-strand linkage and branch points, is also a substrate for endo-1,3-β-glucanase, but its activity is low. Thus, the β-1,6 linkage may be present in marennine, although further investigation is needed to confirm this hypothesis. Although the presence of β-1,3 glucan in marennine is proven in this study, the puzzle of the entire structure of marennine is still elusive, especially on the blue part. Further investigation is ongoing to explain the biological activity of marennine at the molecular level.

## 4. Materials and Methods

### 4.1. Computational Study

#### 4.1.1. Model Construction

The helical chain of laminarin has 20 glucose units. The structure parameters of the model (phi and psi angle) were based on the X-ray crystal structure [50]. The model of laminarin was constructed with Amber for GLYCAM [51]. Since the glucose ring adopts the chair conformation at moderate temperatures, the conformation of the polysaccharide can be described by two dihedral angles and one bridge angle about the glycosidic bond. These parameters are defined as ϕ(H1-C1-O3′-C3′) and ψ(C1-O3′-C3′-H3′). Aniline blue was obtained from Pubchem (CID 135871623).

#### 4.1.2. Molecular Docking

Molecular docking of aniline blue on the triple-helix laminarin was conducted using AutoDock Vina. The exhaustiveness parameter used was 8. The grid box size was 30 Å × 30 Å × 60 Å to cover the whole structure of laminarin.

#### 4.1.3. Molecular Dynamics (MD) Simulation and Binding Energy Calculation

The MD simulation was carried out in aqueous conditions using periodic boundary conditions at the NTP ensemble with the TIP3P water model. The laminarin–aniline blue complex was placed at the center of a periodic box and solvated to a distance of 10Å. The AMBER ff14SB with the GLYCAM06 parameter set for oligosaccharide was used throughout the study. The initial structures of the laminarin–aniline blue complex are all completely dry models at absolute zero temperature. The complex was then immersed in water at 0 K until 278 K (room temperature) gradually over 60 ps in the NVT ensemble. A harmonic restraint of 5 kcal/mol Å2 on the complex was used in the heating stage. Furthermore, 1 ns of NPT equilibration was performed. In this stage, the harmonic restraints are gradually reduced by 1 kcal/molÅ2 until zero. Finally, the production run in the NPT ensemble was performed for 100 ns. The time step at the production run was 2 fs since the SHAKE algorithm was used. The temperature and pressure were controlled using the Langevin thermostat and the Berendsen barostat, respectively. The pressure was maintained at 1 ps. The collision frequency parameter was set to 1 ps−1. The Berendsen barostat was used to control the pressure. The coupling constant and target pressure parameters were set to 1 ps and 1 bar, respectively. The nonbonded cutoff value was set to 9 Å. Particle Mesh Ewald was activated to treat long-range electrostatics. AmberTools was used to analyze the MD trajectories. The binding energy calculation was performed using the MM/GBSA method incorporated in AmberTools [52].

### 4.2. Materials

The supernatant of *H. ostrearia* containing the extracellular marennine (EMn) was obtained from the Biology Department at Le Mans University. The non-axenic *Haslea ostrearia* strain NCC (Nantes Culture Collection) 495 was cultured at 16 ± 1 °C in a temperature-controlled room at an irradiance of 100 µmol m^−2^ s^−1^ provided by Philips TLD 36 W/965 fluorescent tubes (14 h/10 h, light/dark cycle). The cultures were grown in 500 mL Erlenmeyer flasks containing 250 mL of an autoclaved artificial seawater prepared from a commercial sea salt mix (Instant Ocean, Aquarium Systems^®^, pH 7.6 ± 0.2, salinity 32 ppm, see [53]) with an enrichment solution as described in [54]. The carbohydrate hydrolytic enzymes endo-1,3-β-d-glucanase (Barley), exo-1,3-β-d-glucanase (*Aspergillus oryzae*), lichenase (*Bacillus subtilis*), and endo-1,4-β-galactanase (*Aspergillus niger*), as well as barley β-glucan as control substrates were purchased from Megazyme International Ireland Ltd. (Bray, Ireland). The cellulase cocktail (*Aspergillus niger*) was purchased from Merck (Darmstadt, Germany). The cocktail of enzymes contained α-amylase, pectinase, endo-1,3-β-glucanase, xylanase, β-glucosidase, and protease. Laminarin, as a control substrate for endo-1,3-β-d-glucanase (Barley), was purchased from Sigma Aldrich (St. Louis, MO, USA). Sodium acetate buffer was prepared from sodium acetate trihydrate (Prolabo, Paris, France) and acetic acid glacial (Fischer Chemical, Zürich, Switzerland), while sodium phosphate buffer was prepared from sodium hydrogen phosphate and sodium dihydrogen phosphate (Sigma Aldrich, St. Louis, MO, USA). Bovine serum albumin, congo red, 3,5-Dinitrosalicylic acid (DNS, 98%), phosphate buffer saline (PBS), sodium hydroxide, sodium potassium tartrate, and Tris buffer were purchased from Sigma Aldrich (St. Louis, MO, USA).

### 4.3. Purification of Marennine

The supernatants of *H. ostrearia* containing the marennine were filtered using Whatman glass microfiber filters 1.2 μM. Sodium hydroxide 1 N was gradually added to the filtrate while stirring to obtain a green marennine precipitate followed by centrifugation at 10,000 rpm using an Eppendorf Centrifuge 5804 R at room temperature for 30 min. The colorless supernatant was discarded, precipitates were dissolved in 5% formic acid, and pH was checked with pH universal indicator strips. The marennine solution was violet in color at a pH of approximately 3–4. The remaining salts were removed from the solution with dialysis using a SpectrumLabs dialysis membrane with a 3.5 kDa cutoff. The dialysis was carried out for 3 days, and water was changed twice a day. The dialysate was then evaporated under reduced pressure to obtain a concentrated marennine sample.

### 4.4. The Carbohydrate Hydrolytic Enzymes Assay

#### 4.4.1. Detection of Carbohydrate Contents (DNS Assays)

DNS assays were carried out for the detection of carbohydrate content using the hydrolytic enzymes endo-1,3-β-d-glucanase (Barley), endo-1,3-β-d-glucanase (*Trichoderma* sp.), exo-1,3-β-d-glucanase (*Aspergillus oryzae*), endo-1,4-β-galactanase (*Aspergillus niger*), and cellulase cocktail (*Aspergillus niger)*.

The beta-glucan content in the marennine solutions was assessed using endo-1,3-β-d-glucanase (Barley) and exo-1,3-β-d-glucanase (*Aspergillus oryzae*). Three different experiments were set up for each enzyme in a microplate reader. The total volume of each solution was 370 µL. A two-fold serial dilution in sodium acetate buffer pH 4.5 of each enzyme (20 µL) was incubated with marennine concentrated solution (50 µL) with initial mixing for 60 s followed by the addition of 300 µL of DNS reagent (which contained sodium and potassium tartrate, NaOH, water, and DNS) and further incubated for 15 min at 40 °C for 60 min. The absorbance was measured at 540 nm with the BioRad xMark Microplate Spectrophotometer. Laminarin was used as the control substrate for endo-1,3-β-d-glucanase (Barley) and exo-1,3-β-d-glucanase (*Aspergillus oryzae*). The experiments were conducted in duplicates. Similar experiments were carried out using a cellulase cocktail (*Aspergillus niger)* and Endo-1,4-β-galactanase (*Aspergillus niger*) to observe the content of glucose and galactan in the sample, respectively.

Mixed-linkage β-glucan content was observed using lichenase (*Bacillus subtilis*) with barley β-glucan as a control substrate. A two-fold serial dilution of lichenase was prepared in sodium phosphate buffer pH 6.5, a marennine sample was added, and the solution was incubated for 30 min at 37 °C. Tris buffer 2% was added as a stopping reagent, and the absorbance was monitored at 405 nm.

#### 4.4.2. Thin-Layer Chromatography

Thin-Layer Chromatography (TLC) was performed on the silica Gel GF-254 aluminum plate using butanol, acetic acid glacial, and water with a ratio 2:1:1 as the mobile solvent. The visualization was performed by spraying aniline–diphenylamine in 80 mL acetone and 20 mL conc. HCl. The 0.1% standard solutions were used as controls, and 0.1% enzymes were used as samples.

### 4.5. Nuclear Magnetic Resonance Spectroscopy

A total of 250 μL of endo-1,3-β-d-glucanase (Barley) was added to 7 mL concentrated marennine solution and was incubated at 37 °C for 24 h as a sample preparation before NMR and MS studies. The supernatant was dialyzed with membrane cutoffs of 100–500 Da and 3.5 kDa. The dialysis was carried out for 3 days, and the water was changed twice a day. The solution outside the membrane was evaporated until dry.

All NMR experiments were carried out on a Bruker Avance 400 MHz spectrometer equipped with a 5 mm BBFO+ probe head. Samples were dissolved in 0.5 mL D_2_O with 40 mM NaN_3_ at pD 6.6 (corresponding to pH 7.0) to a concentration of 2.4 mM. The NMR spectra were processed using the MestReNova program.

### 4.6. Mass Spectroscopy

ESI–MS measurements were performed on a MicrOTOF-Q III (Bruker, Billerica, MA, USA) mass spectrometer. Extracellular marennine was diluted to 1 mg mL^−1^ in a 2/8 water–methanol mixture (*v*/*v*) acidified with 0.1% formic acid.

### 4.7. Colorimetric Assay

This colorimetric assay technique was created in response to detecting a particular interaction between congo red reagent and 1,3-β-d-glucan in marennine and commercial laminarin samples. The bathochromic change in congo red’s visible absorption maximum was used to evaluate the interaction [55]. 

A total of 140 µL purified marennine samples and 140 µL 244 µM congo red in 15 mM PBS pH 7.4 were reacted and homogenized in microplate wells. A BioRad xMark Microplate Spectrophotometer was used to record the absorption spectra. The absorption maximums of these spectra were compared with the congo red reagent only. A similar assay was also carried out for the laminarin samples.

## 5. Conclusions

This work aims to make progress in the identification of the chemical structure of marennine using carbohydrate hydrolysis enzymes. The successful hydrolysis of marennine with endo-1,3-β-glucanase indicated that the complex structure contains 1,3-β-glucan possibly in a triple-helix conformer. The NMR spectra of marennine hydrolysate showed traces of mono- and oligosaccharides with the signature of β-glucan. This finding is supported by the mass spectra, which interestingly revealed the consistent fragmentation of 1,3-β-glucan at *m*/*z* 115. The evidence of β-glucan as part of marennine could explain the structural features behind its biological activities, such as antibacterial, antioxidant, anticancer, and antiviral activities. Nevertheless, the blue-colored fragment of marennine is still elusive. Additional simulation work was performed to probe a possible interaction between laminarin, a known triple-helix polysaccharide, and blue dye, i.e., aniline blue. It is hypothesized that the blue chromophore of marennine acts in a similar manner to the carbohydrate backbone. Further analysis should be performed to complete the elucidation of the marennine structure.

## Figures and Tables

**Table 1:** List of *m*/*z* of mass spectroscopy spectrum of marennine after hydrolysis with the mixed enzyme.

*m*/*z*	Res.	S/N	I	Area	*m*/*z*	Res.	S/N	I	Area
338.3423	7858	61.7	3964	198	663.4514	8787	53.3	4257	388
340.2616	7269	53.4	3432	184	707.9951	9313	76.5	6636	625
363.0163	8122	165.8	10,582	541	710.4839	7626	41.7	3621	386
367.9715	8102	169.6	10,784	557	712.9524	9273	162.5	14,136	1269
368.4239	7415	85.9	5460	318	713.9527	8976	40.0	3480	353
369.3569	6621	113.1	7183	536	714.9494	9167	46.0	4008	377
372.9262	8127	80.1	5098	279	715.4331	9081	36.6	3200	299
465.9335	8246	62.1	3940	256	717.9085	9600	107.4	9419	819
478.0092	8300	212.8	13,685	886	722.8635	9379	44.4	3936	357
479.4769	7682	51.1	3287	264	738.6270	8533	76.0	7017	702
480.4846	8295	52.0	3351	251	739.6300	8094	37.2	3438	370
482.9647	8276	222.6	14,382	949	762.6233	8849	42.7	4081	441
484.9620	8455	46.4	3004	203	764.6416	8659	78.8	7577	782
487.9197	8431	116.8	7554	495	765.6435	8608	41.0	3958	417
493.4906	7827	50.9	3276	263	766.6724	6902	37.4	3629	455
507.5080	7881	64.7	4187	344	767.9697	9063	31.7	3081	344
521.5163	6961	48.3	3186	289	768.6959	8135	37.2	3633	415
533.5245	8423	65.0	4339	347	770.4509	9338	36.8	3606	365
535.5224	6200	87.2	5832	579	772.9288	9481	30.7	3026	304
537.9850	8686	65.2	4353	331	780.6974	8191	31.5	3118	400
540.4665	7830	64.5	4319	346	782.7118	8268	36.9	3650	414
550.5512	7702	53.6	3606	334	794.7155	8860	44.4	4443	545
575.9760	8884	48.7	3447	262	796.7300	8753	43.8	4394	536
580.9268	8622	63.8	4586	359	808.7304	8727	40.1	4087	470
585.8814	8629	45.4	3286	267	810.7429	8640	35.6	3635	423
593.0026	8843	202.2	14,650	1158	820.7454	5771	30.1	3093	531
595.0003	8691	51.3	3718	287	822.7517	8179	44.7	4594	579
595.4838	8419	70.5	5117	440	822.9850	9277	39.4	4053	452
597.9588	9052	279.4	20,362	1514	824.7585	8208	33.7	3467	456
598.9578	8835	52.5	3834	313	827.9470	9632	89.7	9298	945
599.9560	8854	66.2	4836	391	828.9453	9355	30.2	3134	347
600.4503	6397	47.8	3498	337	829.9440	9542	31.4	3268	352
602.9137	8932	148.4	10,889	848	832.9015	9696	66.5	6947	713
607.8698	8968	55.7	4109	325	837.8419	8148	35.6	3740	429
652.9772	9018	55.5	4357	397	942.9382	9748	49.8	5284	676
655.4585	8970	61.2	4828	502	947.8961	9786	42.8	4527	551
657.9346	9103	44.5	3516	313					

## Data Availability

All data analyzed during this study are included in this article.

## Supplementary Materials

The following supporting information can be downloaded at: https://www.mdpi.com/article/10.3390/molecules28155625/s1, Figure S1: The ^1^H-NMR spectrum of marennine after enzymatic hydrolysis. The peaks at 3.5 and 4.7 ppm correspond to glycerol and HDO, respectively; Figure S2: The ^13^C-NMR spectrum of marennine after enzymatic hydrolysis. The peaks at 60–75 ppm correspond to glycerol; Figure S3: 2D NOESY spectrum of marennine after enzymatic hydrolysis.

## Sample Availability

Samples of the compounds marennine are available from the authors.
