# Supplementary material for: Theoretical and Experimental Studies on the Evidence of 1,3-β-Glucan in Marennine of Haslea ostrearia
Source: Molecules. 2023 Jul 25;28(15):5625. doi: 10.3390/molecules28155625 (PMC10419454; doi:10.3390/molecules28155625)
Supplement: Supplementary file 1 [file molecules-28-05625-s001.zip › molecules-2477603-supplementary.pdf]

Supporting Information

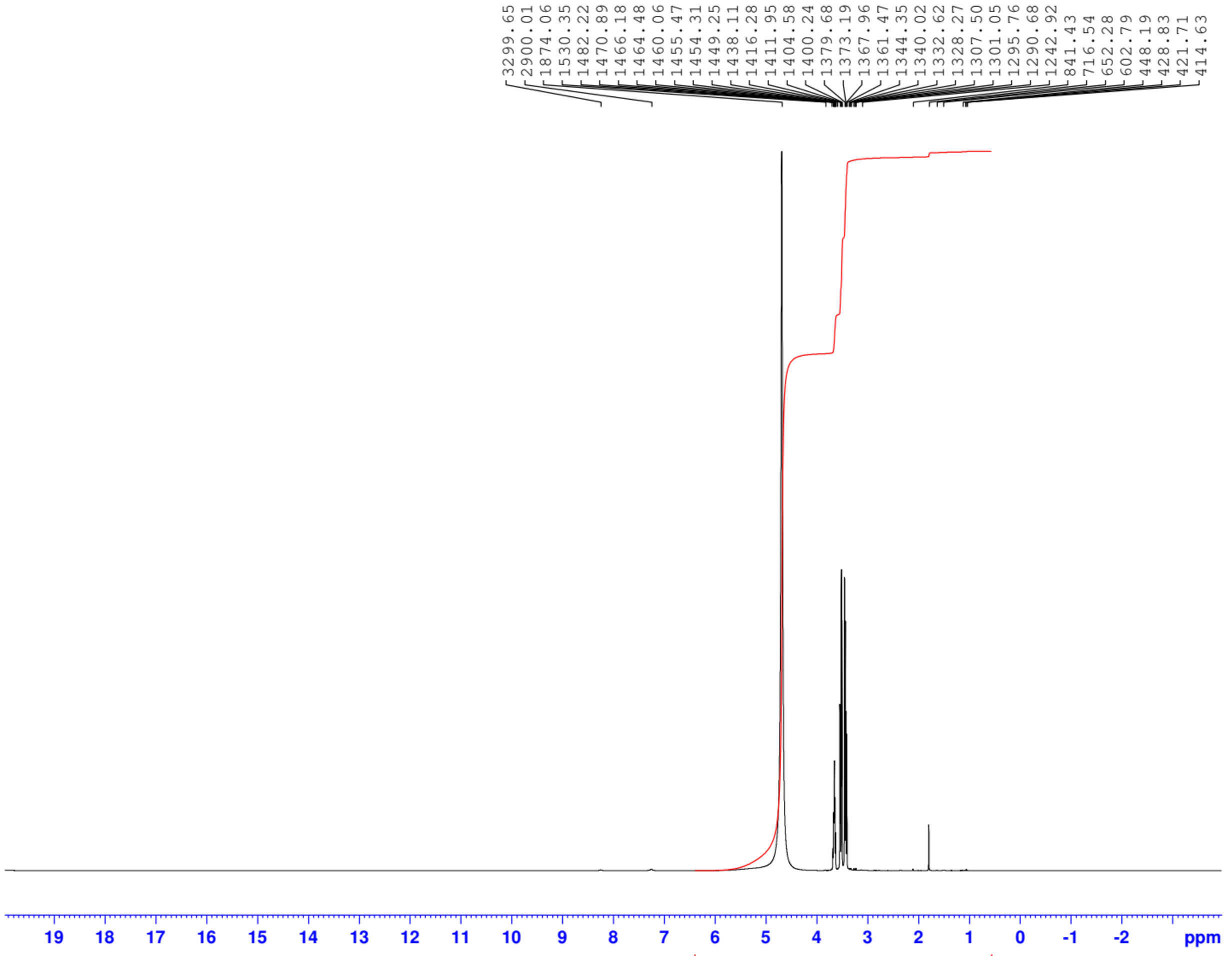

Figure S1. The ^{1}H-NMR spectrum of marennine after enzymatic hydrolysis. The peaks at 3.5 and 4.7 ppm correspond to glycerol and HDO, respectively.

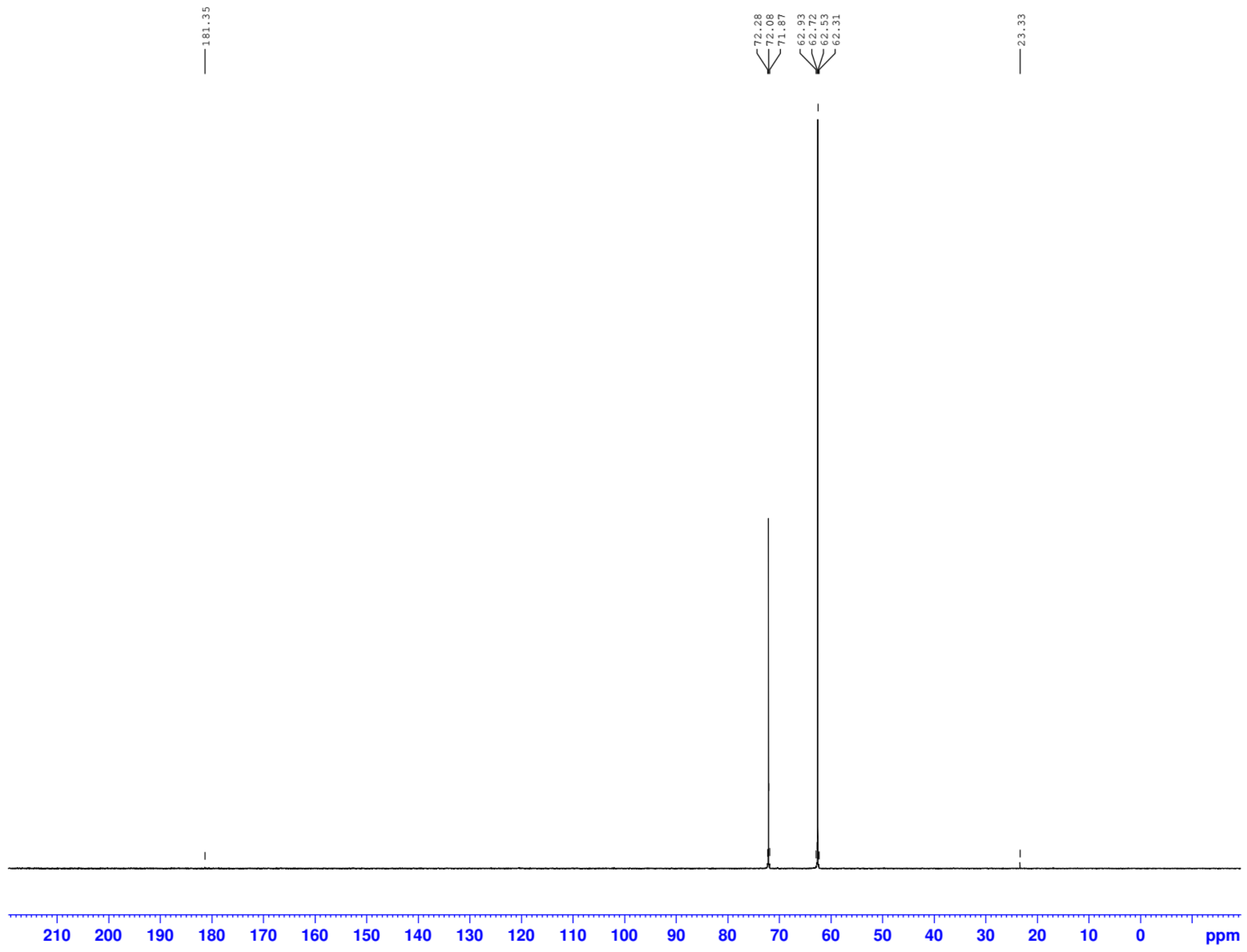

Figure S2. The ^{13}C-NMR spectrum of marennine after enzymatic hydrolysis. Tha peaks at 60 – 75 ppm correspond to glycerol.

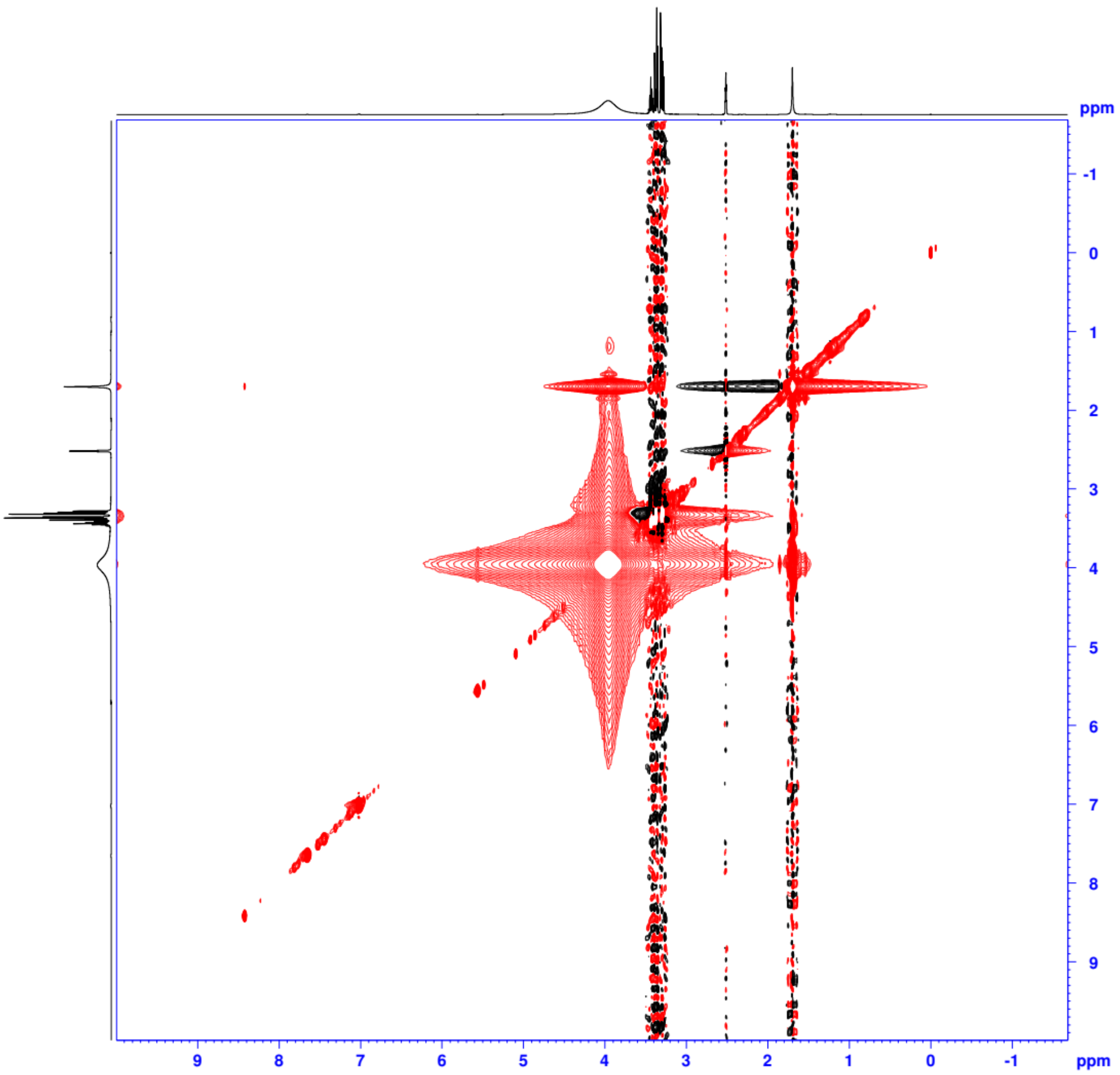

Figure S3. 2D NOESY spectrum of marennine after enzymatic hydrolysis.
